# Probabilistic prediction and context tree identification in the Goalkeeper game

**DOI:** 10.1038/s41598-024-66009-w

**Published:** 2024-07-05

**Authors:** Noslen Hernández, Antonio Galves, Jesús E. García, Marcos D. Gubitoso, Claudia D. Vargas

**Affiliations:** 1 INTHERES, Université de Toulouse, INRAe, ENVT, Toulouse, France; 2 Instituto de Matemática e Estatística, Universidade de São Paulo, São Paulo, Brazil; 3 Universidade Estadual de Campinas, Campinas, Brazil; 4 Instituto de Biofísica Carlos Chagas Filho, Universidade Federal do Rio de Janeiro, Rio de Janeiro, Brazil

**Keywords:** Statistical learning, Probabilistic sequences, Context tree models, Probability matching, Probability maximizing, Decision, Learning algorithms, Decision

## Abstract

In this article we address two related issues on the learning of probabilistic sequences of events. First, which features make the sequence of events generated by a stochastic chain more difficult to predict. Second, how to model the procedures employed by different learners to identify the structure of sequences of events. Playing the role of a goalkeeper in a video game, participants were told to predict step by step the successive directions—left, center or right—to which the penalty kicker would send the ball. The sequence of kicks was driven by a stochastic chain with memory of variable length. Results showed that at least three features play a role in the first issue: (1) the shape of the context tree summarizing the dependencies between present and past directions; (2) the entropy of the stochastic chain used to generate the sequences of events; (3) the existence or not of a deterministic periodic sequence underlying the sequences of events. Moreover, evidence suggests that best learners rely less on their own past choices to identify the structure of the sequences of events.

## Introduction

The aim of this work is to model the performance of a player trying to guess successive choices displayed by an electronic video game called the Goalkeeper game (https://game.numec.prp.usp.br/). In this game, playing the role of a goalkeeper, the participant has to guess at each trial the next direction to where the penalty kicker will send the ball. An animation feedback then shows to which direction the ball was actually sent. The sequence of kicks is selected by a stochastic chain with memory of variable length.

Stochastic chains with memory of variable length were introduced by Rissanen^1^ as a universal model for data compression. Rissanen observed that, very often in experimental datasets composed by sequences of symbols, each new symbol appears to be randomly selected by taking into account a sequence of past units whose length is variable and changes as a function of the sequence of past units itself. Rissanen called *context* the smallest sequence of past symbols required to generate the next symbol. The set of contexts can be represented by a rooted and labeled oriented tree, henceforth called *context tree*. The procedure to generate the sequence of symbols is defined by the context tree and an associated family of transition probabilities used to choose each next symbol, given the context associated to the sequence of past symbols at each time step. From now on, stochastic chains with memory of variable length will be called context tree models. Under suitable continuity conditions, stationary stochastic chains can be well approximated by a context tree model^2^. For that reason, they have been largely used to model biological and linguistic phenomena^3–11^. In the experimental protocol considered here the sequences of directions chosen by the kicker have been generated by context tree models.

In the Goalkeeper game, the participant was instructed to stop the penalty kicks. Obviously, the intrinsic randomness of the algorithm used by the kicker to choose the directions makes it impossible to stop all the penalty kicks. However, the full identification of the context tree and the associated family of probability distributions used by the kicker is an important asset to increase the goalkeeper’s success rate. Moreover, adopting a good strategy to face the randomness of the kicker’s choices might maximize the goalkeeper’s success rate.

In the context of probability learning, two strategies have been proposed to address the problem of making correct guesses in sequences produced by independent stochastic chains (i.e., chains in which the generation of a new symbol is not influenced by the past units). In the *matching strategy*, participants match their response probabilities to the actual probabilities of outcomes. In other words, when faced with multiple options, participants who exhibit the matching strategy will distribute their responses in a way that mirrors the likelihood of each option being correct or successful. In the second strategy, called the *maximizing strategy*, participants consistently choose the option with the highest expected probability of success^12–14^.

In our experimental protocol, an extra difficulty appears, namely, the fact that the probability distributions used by the kicker depend on the successive contexts occurring in the sequence of his previous choices. This means that the goalkeeper must deal simultaneously with the problem of identifying the contexts and its associated transition probabilities as well as that of choosing a strategy to respond. When dealing with structured sequences such as context tree models, where the probability of an outcome depends on previous outcomes (i.e., the context associated with past observations), the concepts of matching and maximizing strategies take a different meaning. In this setting, matching corresponds to a strategy where the participant aligns their predictions with the conditional probabilities associated with the context of the sequence of past events, thus emulating the procedure used to generate the sequences of events. Maximizing, on the other hand, involves choosing the option with the highest probability according to the conditional probabilities associated with that context. Both strategies require dynamically updating, at each step, the probabilities assigned to different outcomes, based on the context occurring at that step in the sequence of stimuli. A double problem of this type was already considered by Wang et al.^15^.

In cases where a participant has not yet learned the contexts or dependencies within the sequences, he/she may erroneously infer structures along with their respective probabilities and employ the misinterpreted probabilities to guide his/her decisions, either by matching them or by selecting the option he/she perceives as having the highest probability of success. Furthermore, the participant may rely on simpler strategies that do not involve adapting to the observed data. For example, the participant may randomly select outcomes with equal probability without considering the observed past sequence or contexts. Otherwise, the participant may consistently choose a specific outcome regardless of the observed past sequence or context. These strategies are simplistic and may not perform well in scenarios where the outcomes are determined by the context associated to past symbols. We adopt the term *suboptimal strategies* as a comprehensive descriptor for strategies that are less optimal than matching. This term doesn’t specify a particular type of deviation from optimality but rather functions as a broad category encompassing any strategy that fails to fully align with the observed context-dependent transition probabilities.

In this article we address two related issues. First, which features of the stochastic chain generating the sequences of events make it more difficult to predict. Second, how to model the procedures employed by different learners to identify the structure of sequences of events. This is done through a rigorous statistical procedure to identify both the context tree and the strategy used by the goalkeeper to make his guesses. We collected data from 122 participants, each one playing the role of the goalkeeper against a kicker that used one out of four different context tree models. By analyzing their sequences of responses, we investigate whether they correctly identify the context tree model used by the kicker and which strategy they use to face the randomness of the kicker’s choices.

## Results

**Figure 1:**
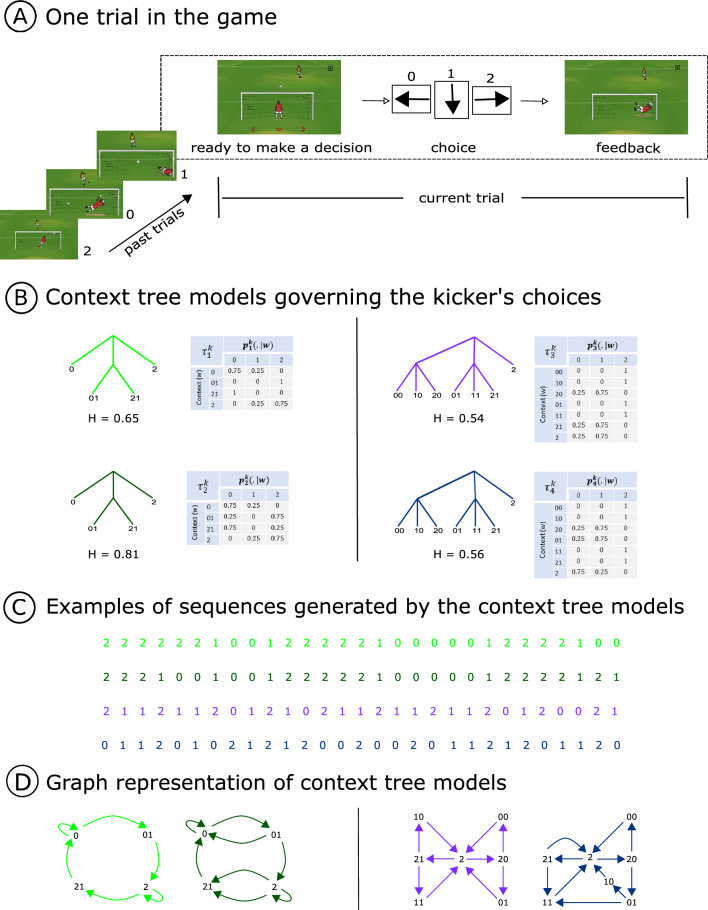
(**A**) Acting as a goalkeeper, the participant must guess, at each step, to where the next penalty kick will be shot by pressing the corresponding keyboard arrow. The options are left, center or right, represented by the symbols 0, 1, and 2, respectively. An animation feedback shows to which direction the ball was effectively sent. (**B**) Context tree models governing the kicker’s choices and their corresponding entropy values. (**C**) Examples of sequences selected by the kicker using each one of the four context tree models. (**D**) Graph representation of the context tree models governing the kicker’s choices.

The aim of the experiment was to model the performance of a player trying to guess successive symbols displayed by an electronic video game called the Goalkeeper game (https://game.numec.prp.usp.br/demo). Playing the role of a goalkeeper, the participant was told to guess one of the three directions to where the kicker could send the ball: left, center, or right, hereafter represented by the numbers 0, 1, and 2, respectively. An animation feedback showed in which direction the ball was effectively sent (Fig. 1A).

The sequences of shot directions were generated by four different context tree models (Fig. 1B). Context tree models are characterized by two elements. The first element is a context tree and the second element is a family of transition probabilities indexed by the leaves of the context tree. In our experimental protocol, the four context tree models characterizing the sequences of the kicker’s choices will be denoted by $\left( \tau ^k_1, p^k_1 \right) ,\left( \tau ^k_2, p^k_2 \right) ,\left( \tau ^k_3, p^k_3 \right) , \left( \tau ^k_4, p^k_4 \right)$. The upper index *k* in the above notation stands for *kicker*. These four context tree models are represented in Fig. 1B. Sequences generated by using each of these context tree models are depicted in Fig. 1C. These context tree models can also be represented as Markovian systems in which the states are defined by the contexts, transiting from one context to another with a certain probability (Fig. 1D).

We first tested whether changing the number of possible outcomes after a given context while keeping fixed the structure of the context tree would affect the learning of these stochastic sequences. This was achieved by associating a stochastic transition probability to contexts that had originally a deterministic one. Therefore, context tree models $\left( \tau ^k_1, p^k_1 \right)$ and $\left( \tau ^k_2, p^k_2 \right)$ were chosen for displaying identical structures but different transition probabilities associated to the contexts 01 and 21 (Fig. 1B, left panel). Changes in these transition probabilities increased the entropy value from 0.65 in $\left( \tau ^k_1, p^k_1 \right)$ to 0.81 in $\left( \tau ^k_2, p^k_2 \right)$. We conjectured that the context tree model with a greater number of stochastic transition probabilities, and consequently, a higher entropy value, would be more difficult to learn.

We were also interested in examining whether the stochastic sequence periodicity would impact the learning process. For this, we fixed the structure of the context tree and modified the associated family of transition probabilities in such a way that, in one case, periodicity would be observed, and in the other, it would not (Fig. 1B, right panel). We conjectured that the context tree model displaying a periodic structure would be easier to learn. Sequences generated by the context tree model $\left( \tau ^k_3, p^k_3 \right)$ can be described as a concatenation of strings 211 in which the symbol 1 is replaced by the symbol 0 with a small probability in an i.i.d way. Exchanging the transition probabilities associated to the contexts 01 and 21, as well as changing the most probable outcome of context 2 disrupted the periodic structure displayed in the context tree model $\left( \tau ^k_3, p^k_3 \right)$ and led to the context tree model $\left( \tau ^k_4, p^k_4 \right)$. These changes were made in such a way that disrupting the periodic structure would not cause any substantial change in the entropy value (0.54 for the context tree model $\left( \tau ^k_3, p^k_3 \right)$ and 0.56 for the context tree model $\left( \tau ^k_4, p^k_4 \right)$.

Finally, comparing the performance obtained with the context tree models $\left( \tau ^k_1, p^k_1 \right)$ and $\left( \tau ^k_2, p^k_2 \right)$ with the ones of the context tree models $\left( \tau ^k_3, p^k_3 \right)$ and $\left( \tau ^k_4, p^k_4 \right)$ would give an indication about if augmenting the number of contexts might increase the learning difficulty. This comparison might also provide new insights into whether entropy is directly related to human learnability.

Data collection was conducted remotely, and compliance measures were incorporated in the videogame to enforce conditions essential for task completion (e.g., the game running in full-screen mode, experiment termination upon mode exit, and participants being disallowed from repeating the experience if they aborted the experiment). We also provided explicit instructions regarding the importance of avoiding distractions during task execution (such as talking with others or using the phone). A total of 122 participants were divided into four groups of 31, 30, 30 and 31, respectively. Each context tree model in Fig. 1B was played by a different group of participants (see section “Methods”). For each participant, a sample was constituted by collecting an ordered sequence of 1000 pairs in which the first element at each pair indicates the choice of the kicker at that step and the second element corresponds to that of the goalkeeper (see Fig. 4A).

### Time evolution of the performance per context tree model

**Figure 2:**
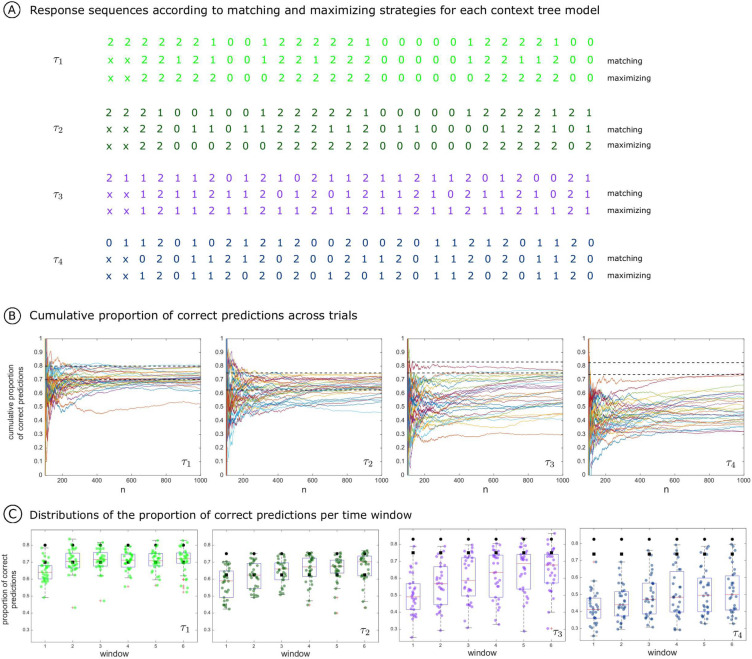
(**A**) Response sequences generated by using the matching and the maximizing strategies for each of the four context tree models used to generate the kicker sequences. (**B**) Time evolution from trial 100 to trial 1000 of the cumulative proportion of correct guesses for each context tree model. The theoretical matching (bottom dashed line) and the theoretical maximizing (top dashed line) scores are indicated. (**C**) Boxplots of proportions of correct guesses across goalkeepers in a sliding window of length 250 pacing at 150 trials for each context tree model. The theoretical matching (black squares) and the theoretical maximizing (black circles) scores are indicated.

Figure 2A shows an example of the sequence generated by each of the four context tree models presented in Fig. 1C and an example of the corresponding response sequence produced by the matching or the maximizing strategies.

For any context tree model, the expected value of the proportion of correct predictions obtained when response sequences are generated according to the matching or maximizing strategies can be theoretically computed (this expected value simply represents the probability of having a match at an arbitrary position). Henceforth, we will refer to the statistical expected value of proportion of correct predictions obtained when the response sequences are generated using the matching and the maximizing strategies as the theoretical matching score and the theoretical maximizing score, respectively.

Figure 2B shows the cumulative proportion of correct predictions across trials per goalkeeper for the four context tree models. Besides, the theoretical matching score and the theoretical maximizing score per context tree model are depicted as dashed lines. An exploratory analysis of the cumulative proportion of correct predictions for models $\left( \tau ^k_1, p^k_1 \right)$ and $\left( \tau ^k_2, p^k_2 \right)$ reveals that the goalkeepers tend to lie mostly between the theoretical matching score and the theoretical maximizing score as the number of trials increases. This is not the case for models $\left( \tau ^k_3, p^k_3 \right)$ and $\left( \tau ^k_4, p^k_4 \right)$.

A sliding window approach was employed to further explore the temporal evolution of the goalkeepers’ performance for each context tree model. Boxplots (Fig. 2C) depict the distributions of the proportions of correct predictions across participants for each time window and each context tree model.

For $\left( \tau ^k_1, p^k_1 \right)$ and $\left( \tau ^k_2, p^k_2 \right)$ the median of the proportion of correct predictions across goalkeepers is above the theoretical matching score from the third time window onward (see the bottom dashed line in Fig. 2B). Also, the interquartile range of proportion of correct predictions for $\left( \tau ^k_2, p^k_2 \right)$ is larger than for $\left( \tau ^k_1, p^k_1 \right)$, suggesting a higher performance variability.

For $\left( \tau ^k_3, p^k_3 \right)$ and $\left( \tau ^k_4, p^k_4 \right)$ the median of the proportion of correct predictions across goalkeepers is smaller than the theoretical matching score in all time windows. In $\left( \tau ^k_3, p^k_3 \right)$, only from the fourth time window onward, the third quartile of the boxplot reaches the theoretical matching score. Results are even worse for $\left( \tau ^k_4, p^k_4 \right)$ as the third quartile is clearly below the theoretical matching strategy score for all windows of analysis.

Finally, there is a much greater variability in the distribution of proportions of correct predictions across goalkeepers in $\left( \tau ^k_3, p^k_3 \right)$ and $\left( \tau ^k_4, p^k_4 \right)$, as compared with $\left( \tau ^k_1, p^k_1 \right)$ and $\left( \tau ^k_2, p^k_2 \right)$.

### Comparative analysis of context tree models’ performance over time windows

To access the differences in performance between the context tree models across time windows, a statistical analysis was done using a repeated measure two-way mixed ANOVA. The intrinsic randomness of each of the context tree models used to guide the choice of the kicker implies that the theoretical maximizing score differs from one model to another (see the top dashed lines in Fig. 2B). Therefore, for statistical analysis, the proportions of correct predictions obtained per goalkeeper and per time window were normalized using the theoretical maximizing score of the corresponding context tree model. These normalized proportions of correct guesses were transformed using a logit transformation (see Supplementary Fig. S1).

To eliminate participants affected by variable compliance, environmental distractions, and other potential factors inherent to the remote context, an univariate linear regression model was fitted to each goalkeeper’s normalized proportions of correct guesses (in logit scale) as a function of the time window. Goalkeepers displaying a negative slope in the estimated regression line were excluded from the subsequent analysis (see Supplementary Fig. S2). As a consequence, the final number of participants per context tree models used in the analysis are 24, 24, 27 and 26, respectively.

The two-way mixed ANOVA analysis of the goalkeepers’ normalized proportions of correct guesses (in logit scale) considers the context tree model as a between subject factor and the time window as a within subject factor. In our case, the levels of the between subject factor were $\left( \tau ^k_1, p^k_1\right) , \left( \tau ^k_2, p^k_2\right) , \left( \tau ^k_3, p^k_3\right) , \left( \tau ^k_4, p^k_4\right)$ and the levels of the within subject factor are 1, 2, 3, 4, 5, 6. A significant interaction between the time window and the context tree model, $F(265.67, 8.22) = 3.04, p=0.003$, indicated that the performance evolved differently across the four context tree models.

**Figure 3:**
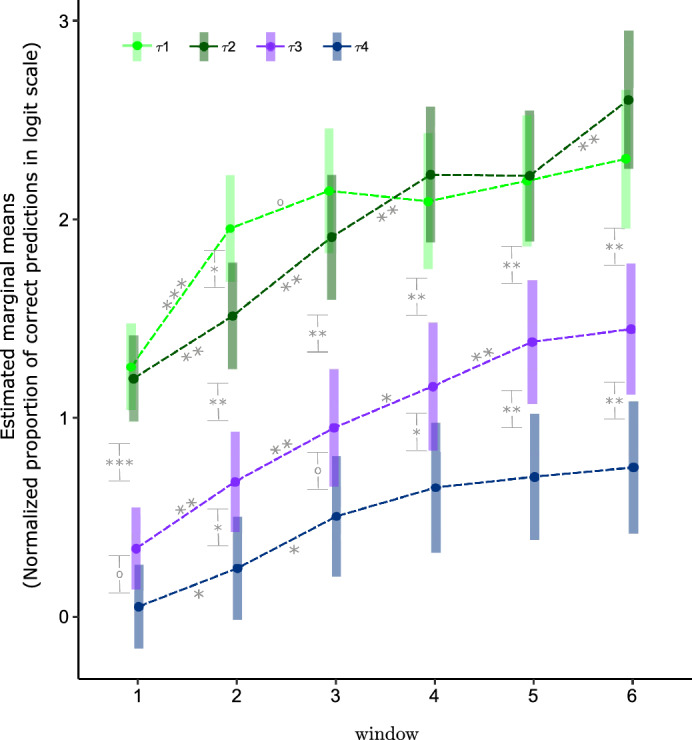
Interaction graph corresponding to the two-way mixed ANOVA analysis using the logit transformation of the normalized proportions of correct predictions as dependent variable and the context tree and time window as factors. Marginal means and 95% confidence intervals of the means are represented with dots and bars, respectively. For each context tree model, the significance level of the difference between successive time windows is indicated using the following convention: $$***$$ for a p-value in the interval [0, 0.0001), $$**$$ for a p-value in the interval [0.0001, 0.01), $$*$$ for a p-value in the interval [0.01, 0.05), $$\circ$$ for a p-value in the interval [0.05, 0.1), null for a p-value in the interval [0.1, 1]. The same convention is used to indicate the significant level of the difference between the means of $$\left( \tau ^k_1, p^k_1 \right)$$ and $$\left( \tau ^k_2, p^k_2\right)$$, $$\left( \tau ^k_2, p^k_2\right)$$ and $$\left( \tau ^k_3, p^k_3\right)$$, and $$\left( \tau ^k_3, p^k_3\right)$$ and $$\left( \tau ^k_4, p^k_4\right)$$, for each time window.

Figure 3 shows the graph of interactions of the two-way mixed ANOVA analysis. The differences between the means at consecutive time windows per context tree model were tested to access the performance evolution for that context tree model. A comparison of the means of the context tree models $\left( \tau ^k_1, p^k_1 \right)$ versus $\left( \tau ^k_2, p^k_2\right)$, $\left( \tau ^k_2, p^k_2\right)$ versus $\left( \tau ^k_3, p^k_3\right)$ and $\left( \tau ^k_3, p^k_3\right)$ versus $\left( \tau ^k_4, p^k_4\right)$ was performed per time window. To globally control the level of significance of the test with multiple comparisons, the Benjamini & Hochberg correction was used^16^.

For model $\left( \tau ^k_1, p^k_1 \right)$, the goalkeepers’ performance strongly improved from the first to the second time window and then stabilized, with no more significant improvement (see Fig. 3 and Supplementary Table S1 for exact p-values). Conversely, for model $\left( \tau ^k_2, p^k_2 \right)$, significant differences appeared up to the fourth time window, then the performance stabilized and presented a significant improvement in the step to the last time window (see Fig. 3 and Supplementary Table S1 for exact p-values). Besides, comparison of $\left( \tau ^k_1, p^k_1 \right)$ and $\left( \tau ^k_2, p^k_2\right)$ performance per time window revealed that the only significant difference occurs at the second time window. Changing the transitions associated to contexts 01 and 21 from deterministic in $\left( \tau ^k_1, p^k_1 \right)$ to random in $\left( \tau ^k_2, p^k_2\right)$ increased the entropy of the corresponding stochastic chains from 0.65 to 0.81. As a consequence, the goalkeepers needed more time to learn the structure of the chain.

For model $\left( \tau ^k_3, p^k_3\right)$, the performance of the goalkeepers improved significantly up to the fifth time window. For $\left( \tau ^k_4, p^k_4\right)$, significant differences were detected only up to the third time window. Besides, $\left( \tau ^k_3, p^k_3\right)$ significantly differed from $\left( \tau ^k_4, p^k_4\right)$ in almost all time windows (see Fig. 3 and Supplementary Table S1 for exact p-values). These results suggest that changes made to model $\left( \tau ^k_3, p^k_3 \right)$ to obtain model $\left( \tau ^k_4, p^k_4\right)$ imposed a high learning difficulty to model $\left( \tau ^k_4, p^k_4 \right)$ in comparison to model $\left( \tau ^k_3, p^k_3\right)$.

Significant differences in performance also appeared between $\left( \tau ^k_2, p^k_2\right)$ and $\left( \tau ^k_3, p^k_3\right)$ for all time windows. Thus, differences in performance can be assumed to occur between $\left\{ \left( \tau ^k_1, p^k_1\right) , \left( \tau ^k_2, p^k_2\right)\right\}$ and $\left\{\left( \tau ^k_3, p^k_3\right) , \left( \tau ^k_4, p^k_4\right) \right\}$.

In conclusion, a comparative analysis of the goalkeepers’ performances across the chosen context tree models revealed that these models display distinct learnability.

### Does the goalkeeper identify the context tree used by the kicker?

**Figure 4:**
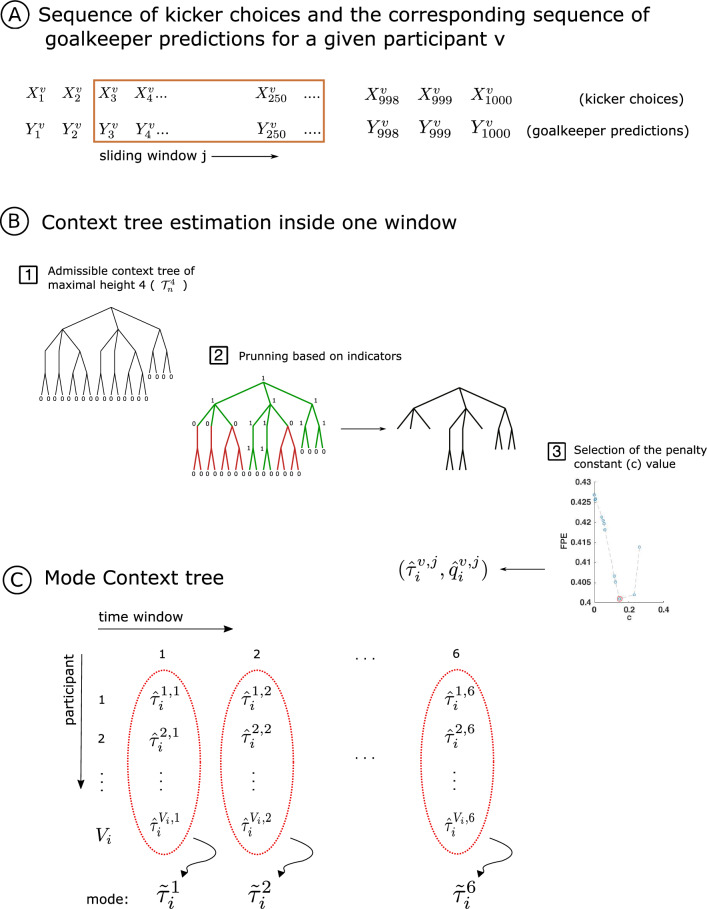
(**A**) For each context tree model and each participant, a sample consisted in an ordered sequence of 1000 pairs of events, each pair corresponding to the successive directions chosen by the kicker and the corresponding guesses of the goalkeeper. (**B1** and **B2**) To retrieve the context tree used by the goalkeeper, we prune the tree of candidate contexts by using a recursive algorithm that solves the BIC criterion (see “Methods” section for more details). (**B3**) The penalty constant in the BIC is chosen so as to minimize the final prediction error risk^3^. (**C**) For each time window, the mode context tree was estimated from the retrieved set of context trees.

We were interested in determining whether it is possible to extract the temporal structure used to generate the sequences of stimuli from the goalkeeper’s responses. This would provide evidence that the goalkeeper is capable of learning the statistical regularities enclosed in those sequences. To achieve this goal, we have introduced a new statistical model selection method that allows estimating both the contexts and the associated family of distributions from each participant’s responses (Fig. 4A).

For each kicker’s model $\left( \tau ^k_i, p^k_i \right) , \ i = 1,\ldots ,4$, to retrieve the structure of the context tree governing the goalkeeper choices, $\left( \hat{\tau }^{v,j}_i, \hat{q}^{v,j}_i\right)$, the model selection procedure was applied separately for each goalkeeper $v \in V$ and each time window $j \in \{1,2,3,4,5,6\}$. The sample then consisted of an ordered sequence of 250 pairs of events (size of the sliding window), each pair corresponding to the successive directions chosen by the kicker and the corresponding guesses of the goalkeeper.

To retrieve the context tree used by the goalkeeper, starting with a tree of candidate contexts (Fig. 4B, Step 1), we prune the tree following a criterion based on indicators computed on each node through an iterative algorithm (Fig. 4B, Step 2). This algorithm seeks the solution for optimizing the Bayesian information criterion (BIC), which defines the estimator. The penalty constant in the BIC is chosen so as to minimize the final prediction error risk^3^ (Fig. 4B, Step 3).

For each context tree model $\left(\tau ^k_i, p_i^k\right)$ and each time window *j*, we end up with a set of trees $\{\hat{\tau }^{v,j}_i, v \in V_i\}$, where $V_i$ is the subset of participants that played against the kicker using the context tree model $\left( \tau ^k_i, p_i^k\right)$ (Fig. 4C). The mode context tree^11^ of this set of trees is computed to summarize the result of the set of goalkeepers.

**Figure 5:**
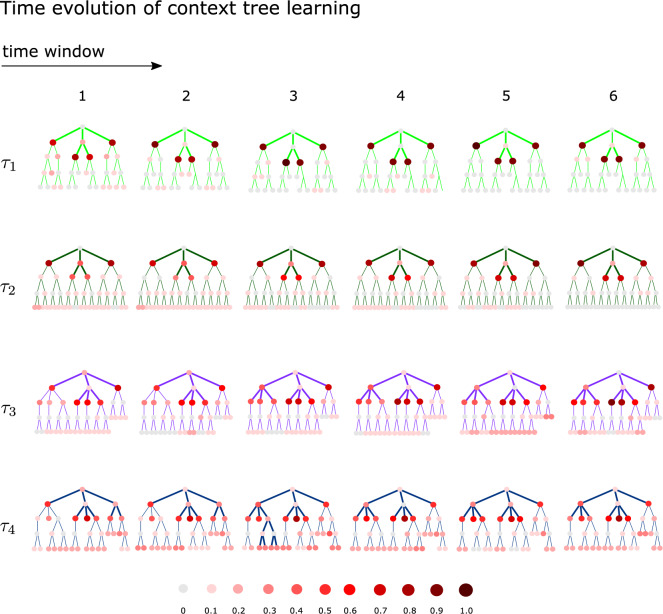
The context trees modeling the goalkeepers’ choices are summarized for each of the four context tree models. To identify these models we used the responses of each goalkeeper and the kicker choices within a sliding window of length 250 pacing at 150 trials. The nodes at each tree structure represent the strings that different goalkeepers identified as a context. Each node is colored from light pink to dark red according to the proportion of goalkeepers identifying that node as a context. Thick lines highlight the mode context tree. The leaves of the mode context tree are the strings that were identified as contexts more often across goalkeepers.

Figure 5 presents the mode context tree computed per time window for each context tree model. This is highlighted in a tree structure that contains all possible past strings up to length 4 that can be identified as a context.

It can be verified that the mode context tree matches that of the kicker’s context tree as early as in the first time window for models $\left( \tau _1^k, p_1^k \right)$ and $\left( \tau _2^k,p_2^k\right)$. Nevertheless, a greater consensus around the contexts used by the kicker is observed in $\left( \tau _1^k, p_1^k\right)$ than in $\left( \tau _2^k,p_2^k\right)$ for all time windows. This suggests that context tree model $\left( \tau _2^k,p_2^k\right)$ is more difficult to learn than context tree model $\left( \tau _1^k, p_1^k\right)$.

For models $\left( \tau _3^k, p_3^k \right)$ and $\left( \tau _4^k,p_4^k\right)$, the mode context tree matches that of the kicker’s context tree in the third and fourth time window, respectively. The fact that a higher number of participants misidentified the kicker’s contexts indicates that these models are more difficult to learn.

### Which strategy the goalkeeper is closer to?

**Figure 6:**
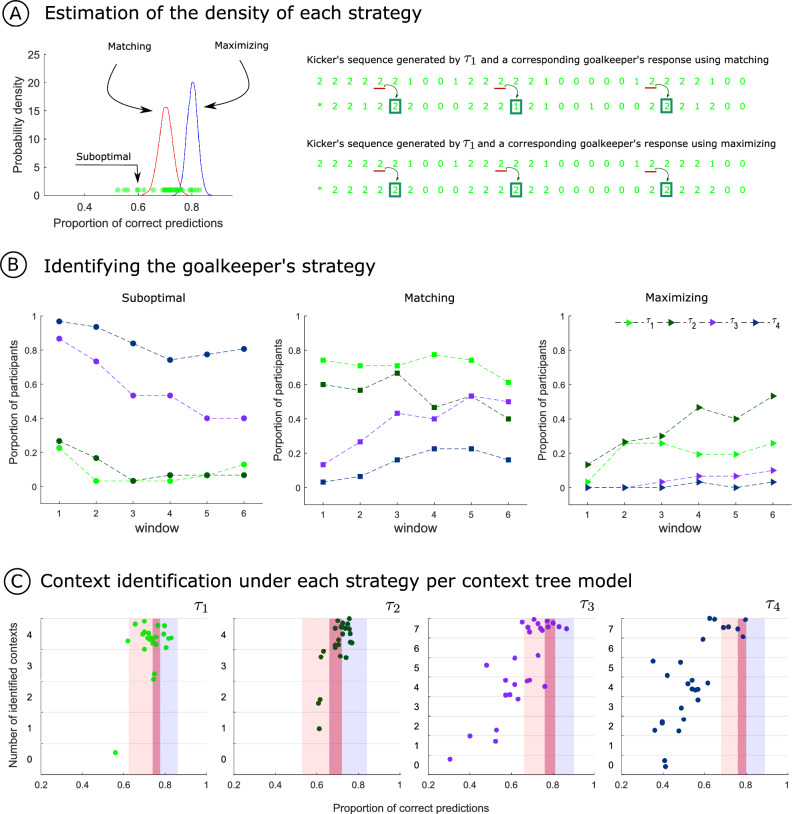
(**A**) Left: The probability density of the proportion of correct predictions for matching and maximizing strategies was estimated using a kernel density estimator on simulated data. For each goalkeeper and each window of analysis, the likelihood that the goalkeeper’s proportion of correct guesses was generated by one of the two estimated distributions (matching, in red vs. maximizing, in blue) is considered to decide to which strategy the goalkeeper is closer to. Right: Example of a kicker’s sequence generated by $\tau _1$ and a corresponding goalkeeper’s response using the matching (upper) and the maximizing (lower) strategies. An example of the response generated by each strategy to context 2 is depicted in red. (**B**) Proportion of goalkeepers per window of analysis that are closer to the suboptimal (left), matching (center) and maximizing (right) strategies per context tree model. (**C**) Number of identified context per goalkeeper as a function of the proportion of correct predictions in the sixth window of analysis for each context tree model . In red, the matching strategy zone, in blue, the maximizing strategy zone and in pink, the superposition of the two strategies.

To identify the strategy to which a given goalkeeper’s performance was closer to, we first estimated, for each context tree model, a probability density of the proportion of correct predictions for the matching and the maximizing strategies. Then, using the two distributions as a benchmark, we compared, for each participant and each window of analysis, the likelihood that the participant’s proportion of correct guesses was generated by one of the two distributions (matching vs. maximizing). If a goalkeeper’s normalized proportion of correct guesses fell below the anticipated lower bound of the matching distribution, we categorized the participant under the suboptimal strategy (see Fig. 6A).

We simulated goalkeeper agents possessing perfect knowledge of the model employed to generate the sequence of kicks. Being aware of the law governing the sequences of kicks, these “fully informed goalkeepers” needed to employ a strategy for generating their response. Consequently, a fully informed goalkeeper employing the matching strategy and another one employing the maximizing strategy were simulated (see Fig. 6A, right panel). The distribution of normalized proportion of correct predictions for each strategy was derived from the response sequences generated by these goalkeepers. This was done by generating 10,000 kicker sequences of size 250 (the size of each window of analysis) and the corresponding response sequences. Then a kernel density estimator was used to obtain a probability density estimate for each strategy.

Figure 6B depicts the proportion of goalkeepers per window of analysis that employed suboptimal, matching, and maximizing strategies per context tree model. For $\left( \tau ^k_1, p^k_1 \right)$ and $\left( \tau ^k_2, p^k_2\right)$ the great majority of participants laid either at the matching or the maximizing strategies in all time windows. Interestingly, for $\left( \tau ^k_2, p^k_2\right)$ the proportion of participants employing the matching considerably reduced in favor of the maximizing strategy across time. For $\left( \tau ^k_3, p^k_3\right)$ most goalkeepers started by employing a suboptimal strategy which was succeeded progressively by a matching strategy. Finally for $\left( \tau ^k_4, p^k_4\right)$ the suboptimal strategy prevailed across time. Almost no goalkeeper achieved the maximizing strategy for $\left( \tau ^k_3, p^k_3\right)$ and $\left( \tau ^k_4, p^k_4\right)$.

In Fig. 6C, the number of identified contexts per goalkeeper was plotted as a function of the proportion of correct predictions for the sixth window of analysis per context tree model. Besides, the range of proportion of correct predictions of the matching (red) and maximizing (blue) strategies was depicted. For all context tree models, the majority of the goalkeepers lying within the matching and the maximizing strategies have identified all the contexts, indicating that, in effect, the goalkeepers who achieve the matching and maximizing scores must learn the contexts.

### Does the goalkeeper rely on his/her own past predictions?

**Figure 7:**
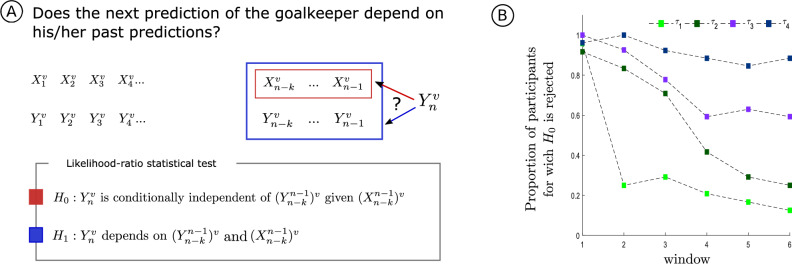
(**A**) Statistical test to verify whether the next goalkeeper’s prediction depends on his/her past choices. (**B**) Proportion of goalkeepers per context tree model across time windows for which the current choice depends both on the past choices of the kicker and on the past choices of the goalkeeper.

The model selection procedure used to retrieve the structures governing the goalkeeper’s choices assumes that he/she takes into account the past events of the kicker’s sequence. To inspect whether the goalkeeper’s predictions are also influenced by their own past choices we performed a statistical hypothesis test. More specifically, we tested the null hypothesis that the goalkeeper’s predictions depend only on the past choices of the kicker by means of a likelihood ratio test (see Fig. 7A and the “Methods”, section Likelihood-ratio statistical tests for independence).

Figure 7B summarizes the results of the hypothesis test. The more the goalkeepers have learned the model, the higher is the probability of not rejecting the null hypothesis, indicating that the goalkeepers stop being influenced by their own past choices. Our data show that most goalkeepers stopped looking at their own past as early as the second window of analysis for the context tree model $\left( \tau ^k_1, p^k_1 \right)$, while for the context tree model $\left( \tau ^k_2, p^k_2\right)$ this happened only from the fourth window of analysis on. Moreover, for the context tree models $\left( \tau ^k_3, p^k_3\right)$ and $\left( \tau ^k_4, p^k_4\right)$, most goalkeepers kept looking at past predictions throughout the game, while trying to identify the tree used by the kicker. This was most evident for the context tree model $\left( \tau ^k_4, p^k_4\right)$. Taken together, these results suggest that the goalkeeper’s decision seems influenced by its own past choices while he/she is still learning the context tree model structure.

## Discussion

We employed a new mathematical framework to model the relationship between a sequence of events generated by four different context tree models and the goalkeeper’s responses. A model selection procedure allowed us to retrieve the context tree and the family of transition probabilities governing the predictions of the goalkeeper. We found that, for a fixed context tree, increasing the number of non-deterministic transition probabilities (and as a consequence, the entropy of the chain) deteriorated the goalkeepers’ performance. Besides, disrupting the periodic structure of the stochastic sequence led to a performance worsening. Retrieving a tree structure and an associated family of transition probabilities from the goalkeepers’ responses indicated which temporal dependencies the goalkeepers have learned. However, this does not imply that the participant has utilized a specific form of context tree model for learning, rather it merely informs the type of temporal dependencies the participants have learned.

We tested whether the goalkeeper’s performance was affected by changing the number of possible outcomes after a given context while keeping fixed the structure of the context tree. For a fixed context tree, the one with a higher number of non-deterministic transition probabilities, and as a consequence, higher entropy $\left( \tau ^k_2, p^k_2 \right)$ was expected to be more difficult to learn. Statistical analysis revealed that participants reached a learning plateau for $\left( \tau ^k_1, p^k_1\right)$ earlier than for $\left( \tau ^k_2, p^k_2\right)$. Besides, most participants stopped looking at their own past as early as the second window of analysis for the context tree model $\left( \tau ^k_1, p^k_1\right)$ while for the context tree model $\left( \tau ^k_2, p^k_2\right)$, this happened only from the fourth window of analysis on. Taken together, these results indicate that for a fixed context tree, a modification in the associated transition probabilities leading to higher entropy values slows down learning.

For a fixed context tree and two different associated families of transition probabilities, namely $\left( \tau ^k_3, p^k_3\right)$ and $\left( \tau ^k_4, p^k_4\right)$, the one that does not display a periodic structure $\left( \tau ^k_4, p^k_4\right)$ was expected to be more difficult to learn comparatively with the one which produces sequences that can be seen as a concatenation of the same deterministic sequence with three elements 211, in which the symbol 1 can be replaced by a new symbol 0 in an i.i.d. way. Indeed, learning rates were lower for the context tree model $\left( \tau ^k_4, p^k_4\right)$ as compared to the context tree model $\left( \tau ^k_3, p^k_3\right)$. Furthermore, the mode context tree of the goalkeeper matched that of the kicker’s from the third window of analysis on for the context tree model $\left( \tau ^k_3, p^k_3\right)$, and from the fourth window of analysis on for the context tree model $\left( \tau ^k_4, p^k_4\right)$. For both context tree models, most goalkeepers kept looking at past predictions throughout the game while trying to identify the tree used by the kicker. This was most evident for the context tree model $\left( \tau ^k_4, p^k_4\right)$.

Finally, the comparison of the performances obtained with the context tree models $\left( \tau ^k_1, p^k_1\right)$ and $\left( \tau ^k_2, p^k_2\right)$ with the context tree models $\left( \tau ^k_3, p^k_3\right)$ and $\left( \tau ^k_4, p^k_4\right)$ might indicate that augmenting the number of contexts increases the learning difficulty. Accordingly, the context tree models $\left( \tau ^k_3, p^k_3\right)$ and $\left( \tau ^k_4, p^k_4\right)$ display much lower learning rates than those of the context tree models $\left( \tau ^k_1, p^k_1\right)$ and $\left( \tau ^k_2, p^k_2\right)$. To sum up, our experimental results show that entropy is not directly related to learnability. We recall that the entropies of models $\left( \tau ^k_1, p^k_1\right)$ and $\left( \tau ^k_2, p^k_2\right)$ are both larger than the entropies of models $\left( \tau ^k_3, p^k_3\right)$ and $\left( \tau ^k_4, p^k_4\right)$, while the first pair is easier to learn than the second one. A non-linear relationship between entropy and complexity of Markov chains has already been pointed out^17^. Besides, alternative measures of complexity for stochastic chains and their relationship with entropy have been proposed^18,19^.

A performance worsening was observed when the number of contexts used by the kicker increased. This is compatible with the findings of Wang et al.^15^, which investigated the dynamics of structure learning by tracking human responses to temporal sequences that change in structure without the participant’s knowledge. In that study, the participants were asked to predict the upcoming item following a probabilistic sequence of symbols, ranging from independent transitions to context-based statistics. The participants succeeded in extracting the behaviourally relevant context length and transition probabilities corresponding to the structure of the presented sequence. Besides, increasing the context length worsened the performance.

In another line of evidence, Kahn et al.^20^ demonstrated that the graph architecture underlying probabilistic sequences fundamentally constrains learning. In their experiment, participants performed a probabilistic motor sequence task in which the order of button presses was determined by the traversal of different graphs (i.e., modular, lattice and random). Reaction times were shown to augment from modular to lattice and random graphs, indicating that probabilistic sequences learning is influenced by the graph topology. For instance, Kahn et al.^20^ found that the inclusion of additional edges (with the same set of nodes) leads to an increase in reaction time. This is equivalent to the transformation done in the graph representation of $\left( \tau ^k_1, p^k_1 \right)$ to $\left( \tau ^k_2, p^k_2\right)$ (see Fig. 1D), where we found a decrease in the proportion of correct guesses. Also, in Kahn et al.^20^ the graph modular structure was associated with lower reaction times as compared to lattice and random graph structures. Likewise, in the present study the graph representation of the context tree model $\left( \tau ^k_3, p^k_3 \right)$ displays a modular structure that relates to the periodicity present in the sequences it generates. This modular structure was disrupted in the context tree model $\left( \tau ^k_4, p^k_4\right)$ (see Fig. 1D). As in Kahn et al.^20^ the breaking of this modular structure could explain the worse performance found in $\left( \tau ^k_4, p^k_4 \right)$ as compared to $\left( \tau ^k_3, p^k_3\right)$.

In probability learning tasks, participants often start from a strategy of random guessing and may then slowly approach the maximizing behavior. Based on an extensive review^13^, Montag concluded that in simple probability learning tasks, participants normally tend to overshoot true matching behavior. In our experimental protocol, the probability distributions used by the kicker depended on the successive contexts occurring in the sequence of his previous choices. In other words, the goalkeeper had to deal simultaneously with the problem of identifying the contexts and its associated transition probabilities as well as that of choosing a strategy to respond. Comparing the performances obtained in $\left( \tau ^k_1, p^k_1\right)$ and $\left( \tau ^k_2, p^k_2\right)$ we observed that more participants went closer to the maximizing strategy in $\left( \tau ^k_2, p^k_2\right)$ than in $\left( \tau ^k_1, p^k_1\right)$. This is consistent with the findings of Wang et al.^15^ showing that participants get closer to maximizing when the complexity of the task increases by changing the order of the stochastic chain from 0 to 1 and 2. In our case, this effect was observed after changing the transition probabilities associated to the contexts 01 and 21 that increased the entropy values associated to model $\left( \tau ^k_2, p^k_2\right)$. Since the maximizing strategy was very rarely found in the case of the context tree models $\left( \tau ^k_3, p^k_3\right)$ and $\left( \tau ^k_4, p^k_4\right)$ the interplay between difficulty of the task and maximizing seems not linear.

The maximizing strategy involves selecting the most probable option based on the distribution of each context, while the matching strategy refers to making predictions directly corresponding to the observed probabilities associated with each context. When a goalkeeper’s response pattern aligns with these definitions, this will be an indication that the goalkeeper is following one of these strategies. If so, the “real” goalkeeper achieves a performance level similar to a fully-informed maximizing (or matching) goalkeeper. In the present work, such alignment was verified through the analysis of the distributions of the proportion of correct predictions. It is important to note that a goalkeeper who emulates (or who chooses the most probable option) based on a misspecified context may not be following the matching (nor the maximizing) strategy.

Additionally, it appears unlikely that goalkeepers who have not yet learned the correct contexts (or at least a significant portion of them) and some key features of their associated distributions (such as the mode) will exhibit the maximizing behavioral pattern. Without this knowledge, they may lack the necessary understanding about the sequence of kicker’s choices to assess potential outcomes and select the option with the highest expected value. A similar line of reasoning can be applied to the matching behavioral pattern. Two interconnected questions arise: Can a goalkeeper who has not learned the contexts and their associated distributions demonstrate matching behavior in their responses? Is it possible for this matching behavior pattern to emerge ‘by chance’ or as a result of employing other heuristics? To answer this question, we have shown in Fig. 6C that the majority of goalkeepers exhibiting the matching behavior have indeed learned most of the contexts. The same rationale applies to the maximizing strategy. Therefore, we argue that effective implementation of these strategies necessitates prior learning or information acquisition. This is in line with the rationale employed in studies in which participants are trained beforehand to learn the distribution of outcomes or where this distribution is explicitly provided before accessing their strategy^21–24^.

Plonsky et al.^25^ proposed that, when it comes to the behavioural phenomena associated with the tendency to rely on small samples, the exploitation of environmental regularities would be more effective than the reliance on more recent experiences. The authors suggest that an optimal strategy can be achieved by employing contingent average rules based on the last *k* outcomes (CAB-k). In the case of the goalkeeper game, each context corresponds to the smallest final string of past symbols containing all the information required to predict the next symbol^10^. Context tree models can be seen as a dynamic environment scenario in which the states are defined by the contexts, transiting from one context to another with a certain probability (see Fig. 1D). Once the participant has learned the contexts, he/she is able to identify the current state of the environment and choose an appropriate strategy that maximizes positive feedback. Therefore, when employing context tree models, the *k* value remains variable. Further work could be done by generalizing CAB-*k* rules in such a way that they can detect contexts of variable length.

Online data collection offers a wide range of benefits, including access to larger and more diverse populations. Comparing performance in a spatial memory task using data from a controlled lab-based setting and from an unsupervised online sample, Segen et al.^26^ found that although the data collected in a conventional laboratory setting and those collected online produced very similar results, the online data was more variable with standard errors about 10% larger than those of the data collected in the lab. In another line of evidence, the variability of crowdsourced behavioral motor data is higher in online large sample sizes as compared with typical in-lab experiments, most likely due to the more heterogeneous pool of participants and a less controlled setting^27,28^. Furthermore, high dropout rates have been consistently reported in online experimental studies^29^. Bönstrup et al.^27^ reported a high exclusion rate percentage in a crowdsourced experiment in which mechanisms of sequence learning were investigated online. Consistent with previous findings showing a higher rate of data exclusion in online experiments compared to traditional in-lab studies, a portion of goalkeepers per context tree model were excluded from our analysis due to indications of task misunderstanding, inattention or negligence in their performance. In conclusion, online data collection allows a higher number of participants and has been recognized as a very useful environment to study motor learning^27^. Notwithstanding, strategies to address higher inter-subject variability and outlier cases become mandatory when it comes to online settings.

In conclusion, our mathematical model assumes that the goalkeeper considers only the past choices of the kicker. This assumption is statistically supported by the data, once the goalkeeper has learned the model. Conversely, our data show that the goalkeeper’s decision is influenced by its own past choices when he/she is still learning the context tree model structure. To the best of our knowledge, this is a new result that express an important feature of the goalkeeper’s learning strategy.

## Methods

### Participants

A total of 122 healthy volunteers (60 female, mean age 31.95, standard deviation 9.41, right handedness 89.34%, all of them with at least the secondary school level and only 6.56% with no game familiarity) were recruited for the experiment. The participants signed an informed consent term after the objective of the study was explained to them. This experimental protocol was approved by the ethics committee of the Institute of Neurology Deolindo Couto at the Federal University of Rio de Janeiro (Plataforma Brasil process number CAAE 58047016.6.1001.5261, statement 259 approval number 1.846.941). We hereby confirm that all research was performed in accordance with the Declaration of Helsinki, and that informed consent was obtained from all participants and/or their legal guardians.

### Experimental protocol

The experiment was conducted remotely and consisted of two steps. In the first step, participants received a link containing an electronic form asking about gender, age, educational level, handedness and familiarity with electronic games, to characterize the sample. After completing this form, the participants received a second e-mail containing the link to access the game.

The game started with an instruction screen with the following statement: “You are a goalkeeper and you must predict before each kick in which region of the goal the ball will be kicked. The penalty taker may kick the ball toward the left, the center or the right of the goal. Your task is to defend the maximum of penalties. Attention, goalkeeper: the penalty taker is not influenced by your choices. During the experiment use the left, down and right arrows on the keyboard to choose the left, center and right side of the goal, respectively. During the game you will have two rest breaks that will be informed. Have fun and improve your performance. The more penalties you defend, the better you will be ranked.”

Four different context tree models were chosen to generate the sequences of kicker’s choices (Fig. 1B). Each participant was assigned to one of the four context tree models. As a result, the four groups consisted of 31, 30, 30 and 31 participants, respectively. Furthermore, within a context tree model group, each participant was exposed to a putative different sequence of kicker’s choices.

For each participant and each context tree model, a sample was constituted by collecting an ordered sequence of 1000 pairs, indicating the successive directions chosen by the kicker and the corresponding guesses of the goalkeeper.

### Structure of the sequence of stimuli

The sequences of directions chosen by the kicker were generated by using a context tree model. A context tree model is characterized by a context tree representing the set of minima suffixes of the different sequences of past required to generate the next symbol. Associated to each context there is a transition probability indicating the probability with which the next symbol is chosen, given the context. For more details on context tree models we refer the reader to Rissanen^1^, Buhlmann et al.^3^ and Galves et al.^30^.

In our experimental protocol, the four context tree models used to generate the sequences of the kicker’s choices will be denoted by $\left(\tau^k_1, p^k_1\right)$, $\left(\tau^k_2, p^k_2\right)$, $\left(\tau^k_3, p^k_3\right)$ and $\left(\tau^k_4, p^k_4\right)$. In the above notation, the upper index *k* stands for *kicker* (Fig. 1B).

The four context tree models are organized in pairs. In each pair the two models have the same context tree but with different associated families of transition probabilities (Fig. 1B).

For the first pair ($\left(\tau^k_1, p^k_1\right)$ and $\left(\tau^k_2, p^k_2\right)$), the changes were done in the transition probabilities associated to contexts 01 and 21. In $\left(\tau^k_1, p^k_1\right)$, the transitions associated to these contexts are deterministic. In opposition, in $\left(\tau^k_2, p^k_2\right)$ we have two possible outcomes: with a small probability (0.25) we return to the same symbol which occurred before symbol 1, and with a high probability (0.75) we change to a new symbol. These changes increase the entropy from 0.65 for model $\left(\tau^k_1, p^k_1\right)$ to 0.81 for model $\left(\tau^k_2, p^k_2\right)$.

In the second pair ($\left(\tau^k_3, p^k_3\right)$ and $\left(\tau^k_4, p^k_4\right)$), sequences generated by $\left(\tau^k_3, p^k_3\right)$ can be described as a concatenation of strings 211 in which the symbol 1 can be replaced by the symbol 0 with small probability (0.25) in an i.i.d way. Conversely, the sequences generated by $\left(\tau^k_4, p^k_4\right)$ can not be obtained by replacing symbols in a concatenation of deterministic strings in an i.i.d way (Fig. 1C). This was achieved by interchanging the transition probabilities associated to contexts 01 and 21 and by changing the most probable outcome in the transition probability associated to context 2. These changes hardly changed the entropy (0.54 for model $\left(\tau ^k_3, p^k_3\right)$ and 0.56 for model $\left(\tau ^k_4, p^k_4\right)$).

### Statistical analysis

In our experiment, the set of participants *V* is divided in four groups according to the kicker’s context tree model $\left(\tau^k_i,p^k_i\right)$, $i=1,..,4$ that each goalkeeper is exposed to (see Fig. 1). For each goalkeeper $v = v(i) \in V$, $X_1^{v}, X_2^{v},\ldots ,X_{n}^{v}$ denotes the sequence of kicker choices and $Y_1^{v}, Y_2^{v},\ldots ,Y_n^{v}$ the goalkeeper’s responses recorded during the exposure to $(X^{v})_n$.

In order to assess the temporal evolution of the performance, most of the statistical analysis was done using a sliding window approach. In this approach a window is formed over 250 trials, and this window slides over the data (150 trials). Therefore we end up with six time windows. We denote by $\left(X_1^{v,j}, Y_1^{v,j}\right),\ldots ,\left(X_m^{v,j}, Y_m^{v,j}\right)$ the data corresponding to the kicker’s choices and goalkeeper predictions for the participant $v \in V$ in the time window *j*, $j = \{1,\ldots ,6\}$.

#### Entropy rate computation for context tree models

Each context tree model has an associated entropy value (Fig. 1B), calculated as follows.

For a stationary time-invariant Markov Chain $(Z_n)_{n}$, with stationary distribution $\varvec{\mu }$, the entropy rate is given by

1 $$\begin{aligned} H(Z) = \lim _{n \rightarrow \infty } H(Z_n|Z_{n-1},\ldots ,Z_1)= & {} \lim _{n \rightarrow \infty } H(Z_n|Z_{n-1}) \nonumber \\= & {} H(Z_2|Z_1) \nonumber \\= & {} \sum _i \mu _i \left( -\sum _j M_{ij}\log M_{ij} \right) , \end{aligned}$$

with *M* the transition matrix.

The stationary distribution is the solution of the equation

2 $$\begin{aligned} \varvec{\mu } M = \varvec{\mu }, \end{aligned}$$

with the additional constrain that $\Vert \varvec{\mu }\Vert = 1$. Equation (2) can be written as $M^T \varvec{\mu }^T = \varvec{\mu }^T$. So, $\varvec{\mu }^T$ is the (normalized) eigenvector of $M^T$ associated to the eigenvalue 1.

To compute the entropy rate of a context tree model $(X_n)_{n}$ compatible with $(\tau , p)$ (for finite $\tau$), we fist compute the representation of $(X_n)_{n}$ as a Markov chain of order $L = \max \{l(w): w \in \tau \}$. And then, transform that Markov chain of order *L* to a first-order Markov chain taking values in the state space $S \subseteq A^L$. The following steps describe the procedure,

1. Compute the set of all states *S*, that is to say the set of all *possible* pasts of length *L* (i.e., all strings of length *L* with probability greater than zero).
2. Compute the transition probability matrix *M*. Note that $M_{ij}$ is the probability of going from the state $s_i$ to the state $s_j$, then it states
  3 $$\begin{aligned} M_{ij} = \left\{ \begin{array}{ll} p\left( {s_j}^{-1}_{-1}|c_{\tau }(s_i)\right) &{} \text{ if } {(s_j)}^{-2}_{-l(s_j)} = {(s_i)}^{-1}_{-l(s_i)+1} \\ 0 &{} \text{ otherwise } \end{array} \right. \end{aligned}$$
  where $c_{\tau }(s_i)$ is the context function that gives the context associated to $s_i$.
3. Using Eqs. (2) and (1) compute the stationary distribution and the entropy.

#### Proportion of correct predictions

The proportion of correct predictions (PCP) is defined as the proportion of times that the goalkeeper prediction matches the choice of the kicker in a defined set of trials *T*. Formally,

4 $$\begin{aligned} PCP_v = \frac{1}{|T|}\sum _{t=1}^{|T|} 1_{\{Y_t^{v} = X_t^{v}\}}, \end{aligned}$$

where |*T*| refers to the cardinal of the set *T*.

The normalized proportion of correct predictions for a participant $v = v(i)$ computed in the set of trials *T* is defined as the proportion of correct predictions on *T* divided by the theoretical maximizing score for the kicker context tree model *i*.

When both the proportion of correct predictions and the normalized proportion of correct predictions are computed across time windows, |*T*| refers to the trials encompassed in the window.

The curve of cumulative proportion of correct predictions corresponding to a goalkeeper *v* is obtained by computing on each trial *t* ($t \in \{1,\ldots ,1000\}$) the proportion of correct predictions considering the set $T = \{1,\ldots ,t\}$

5 $$\begin{aligned} CPCP_v(t) = \frac{1}{t}\sum _{m=1}^{t} 1_{\left\{ Y_m^{v} = X_m^{v}\right\} }. \end{aligned}$$

#### ANOVA

Let $z_{ijv}$ be the normalized proportion of correct predictions of the goalkeeper *v* playing the kicker’s context tree model $\left(\tau_i^k, p_i^k\right)$ in the time window *j* after a logit transformation.

An univariate linear regression model was fitted to each goalkeeper’s normalized proportions of correct guesses $z_{ijv}$ as a function of time window *j*, i.e., $z_{ijv} = \gamma _{v}*j + b_v$. Goalkeepers with negative slope $\gamma _{v}$ were excluded from the statistical analysis (see supplementary material for more details).

The fitted mixed ANOVA model is,

6 $$\begin{aligned} z_{ijv} = \mu + \alpha _j + \beta _i + (\alpha \beta )_{ij} + s_{(v(i))} + e_{ijv}, \end{aligned}$$

where $\mu$ is the overall mean, $\alpha _j, j=1,\ldots ,6$ is the effect of the *j*th-level of the within subject factor (time window), $\beta _i, i=1,\ldots ,4$ is the effect of the *i*th-level of the between subject factor (kicker’s context tree model), $(\alpha \beta )_{ij}$ is the *ij* interaction effect, $s_{(v(i))}$ is the random effect of the *v*th goalkeeper and $e_{ijv}$ is a random noise.

#### Statistical model selection procedure

For each goalkeeper $v \in V$ and each time window $j \in \{1,\ldots ,6\}$ the data $\left(X_1^{v,j}, Y_1^{v,j}\right),\ldots ,\left(X_n^{v,j}, Y_n^{v,j}\right)$ is used to estimate the context tree $\hat{\tau }^{v,j}$ and the family of distributions $\hat{q}^{v,j}$ governing the predictions of the goalkeeper (Fig. 4A). This estimate is found by solving an optimization problem using the Bayesian Information Criterion (BIC). Model selection for context tree models via BIC was first addressed in Csiszar and Talata^31^. In this work, we adapted the procedure introduced by Csiszar and Talata^31^ to a sequence of random objects driven by context tree models. That is to say, a pair of sequences in which the first one (the sequence of stimuli) is a context tree model and the second one (the sequence of responses) is assumed to be generated in such a way that a new element of the response sequence is chosen according to the probability measure associated to the context ending at that step in the sequence of stimuli (see Duarte et al.^32^ and Hernandez et al.^33^ for a formal definition of sequence of random objects driven by context tree models).

Given a sample $(X_1, Y_1),\ldots ,(X_n, Y_n)$ and a constant $c>0$, our BIC estimator is defined as

7 $$\begin{aligned} \hat{\tau }_{n;c}=\hat{\tau }\left(\left(X,Y\right)^n_1;c\right)= \textrm{argmax}_{\tau \in \Gamma ^L_n}\Big \lbrace \log L_{\left(\tau ,\hat{q}\right)} - c\cdot \text{df}(\tau )\log (n) \Big \rbrace , \end{aligned}$$

where $\text{df}(\tau)$ stands for the degree of freedom of the model, $L_{(\tau ,\hat{q})}$ for the likelihood (defined later in Eqs. (11) and (12), respectively) and $\Gamma ^L_n$ is the set of all admissible context trees of maximal height *L*. An admissible context tree of maximal height *L* is any irreducible tree of height *L* in which all the contexts are finite sequences of symbols in the alphabet $A=\{0,1,2\}$ that effectively occurs in the sequence of stimuli.

Csizar and Talata^31^ showed that optimization problems of the type of (7) can be solved efficiently through an inductive procedure, which in our case states as:

1. Compute the admissible context tree of height *L* for the sequence of stimuli $(X_1,\ldots ,X_n)$, denoted by $\mathcal {T}_n^L$, where *L* is a suitable positive integer. As an illustration, Fig. 4B, Step 1, shows an admissible context tree of height 4 for a sequence generated by the context tree model $(\tau _3^k,p_3^k)$.
2. For any leave in $\mathcal {T}_n^L$ (i.e, any $w\in \mathcal {T}^L_n$) define
  the quantity $$V_{w,n} = V_w((X,Y)^n_1) = n^{-c\cdot df(w)} L_{(w,\hat{q})}((X,Y)^n_1)$$
  and the indicator $\mathcal {X}_{w,n}=\mathcal {X}_w((X,Y)^n_1)=0$
  Figure 4B, Step 1, shows all the indicators initialized in zero for the leaves of the admissible context tree of height 4.
3. Then, for any internal node of the tree (i.e., any $w\prec u \in \mathcal {T}_n^L$) define recursively
  the quantity
    8 $$\begin{aligned} V_{w,n} = \max \Big \lbrace n^{-c\cdot df(w)} L_{(w,\hat{q})}((X,Y)^n_1)\, , \, \prod _{b\in A}V_{bw,n} \Big \rbrace \end{aligned}$$
  and the indicator
    9 $$\begin{aligned} \mathcal {X}_{w,n} = 1_{\Big \lbrace \prod _{b\in A}V_{bw,n} > n^{-c\cdot df(w) } L_{(w,\hat{q})}((X,Y)^n_1) \Big \rbrace .} \end{aligned}$$
4. The rule to prune the tree and, as a consequence, obtain the solution of (7) can be written as
  10 $$\begin{aligned} \hat{\tau }_{n;c} = \left\{w \preceq s \in \mathcal {T}^L_n: \mathcal {X}_{w,n}=0 \text{ and } \mathcal {X}_{u,n}=1 \text{ for } \text{ all } u\prec w\right\}. \end{aligned}$$
  In words, that means that a node is maintained if it has indicator equal 0 and all the nodes in the path from this node to the root have indicator equal 1. Figure 4B, Step 2, exemplified this criterion.

The following algorithm summarized the statistical procedure.

**Algorithm 1:**
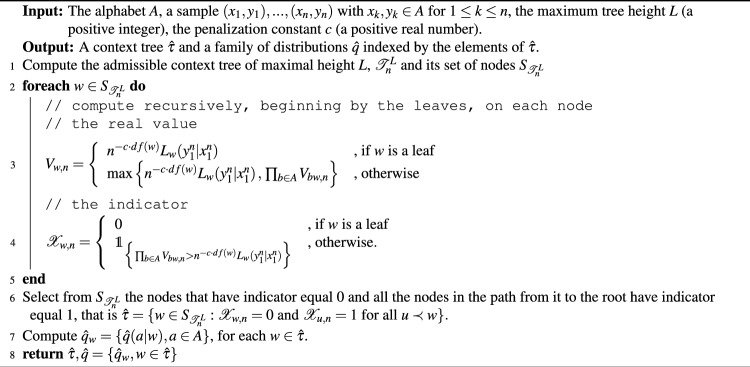
Model selection algorithm for Sequence of random objects driven by context tree models based on BIC

In what follows, we define some of the quantities used in the description of the method and the algorithm:

Degree of freedom of the model:

11 $$\begin{aligned} \text{df}(w)= \sum _{a\in A} 1_{\{N_n^{XY}(w,a)\ge 1\}} - 1, \quad \text{df}(\tau) = \sum _{w\in \tau } \text{df}(w) . \end{aligned}$$

Likelihood of the model:

12 $$\begin{aligned} L_{u}(Y_1^n\mid X^n_1) =\prod _{a \in A} \hat{q}(a|u)^{N_n^{XY}(u,a)}, \quad L_{(\tau ,\hat{q})} = L_{(\tau ,\hat{q})}(Y_1^n\mid X^n_1) =\prod _{u\in \tau } L_{(u,\hat{q})}(Y_1^n\mid X^n_1) \end{aligned}$$

Estimated transition probability of observing the symbol *a* in the response sequence given the past *u* in the stimuli sequence:

13 $$\begin{aligned} \hat{q}(a|u) = \frac{N_n^{XY}(u,a)}{N_n^X(u)} = \frac{N_n^{XY}(u,a)}{\sum _{a'\in A} N_n^{XY}(u, a')} , \end{aligned}$$

where

14 $$\begin{aligned} N_n^{XY}(u,a) = \sum _{t = l(u)}^{n-1}\mathbbm {1}_{ \{ X_{t-l(u)+1}^t = u; Y_{t+1} = a \} }, \end{aligned}$$

and

15 $$\begin{aligned} N_n^X(u) = \sum _{t = l(u)}^{n-1}\mathbbm {1}_{\{X_{t - l(u)+1}^t = u\}}. \end{aligned}$$

It can be seen that there is a penalization constant *c* involves in the optimization criterium. This is a hyperparameter whose value must be specified a priori. Small values of *c* results in big context trees and, consequently, overfitted models while high values of *c* gives rise to context tree of small size and underfitted models. To choose the optimal value of *c* we used a procedure based on Risk functions introduced by Buhlmann et al.^34^. In particular, we used the final prediction error (FPE) risk. Figure 4B, step 3, exemplified the values of FPE as a function of the values of *c* and the selection of the optimal value.

For each kicker’s context tree model $(\tau _i^k, p_i^k)$, we have on each time window *j* the set of context trees $\mathcal {C}_{i,j} = \{\hat{\tau }^{v,j}, v \in V_i\}$, where $V_i$ is the subset of goalkeepers exposed to the kicker’s context tree model $(\tau _i^k, p_i^k)$. We summarize this set of trees by computing the mode context tree $\tilde{\tau }_i^j$ (see Fig. 4C).

#### Likelihood-ratio statistical tests for independence

A question of interest is to know whether the goalkeeper’s prediction $Y_n^v$ at trial *n* is influenced only by the past kicker shootings $\left(X_1^{n-1}\right)^v = \left(X_1^v,\ldots , X_{n-1}^v\right)$ or by both, the past kicker shootings $\left(X_1^{n-1}\right)^v$ and its own past plays $\left(Y_1^{n-1}\right)^v = \left(Y_1^v,\ldots , Y_{n-1}^v\right)$. To address this question, we consider the following testing problem,

$$\begin{aligned} H_0 &: Y_n^v \text{ is conditionally independent of } \left(Y_{n-k}^{n-1}\right)^v \text{ given } \left(X_{n-k}^{n-1}\right)^v \quad \text{vs} \\ H_1 &: Y_n^v \text{ depends on } \left(X_{n-k}^{n-1}\right)^v \text{ and } \left(Y_{n-k}^{n-1}\right)^v, \end{aligned}$$

where *k* refers to the length of the past considered in the sequences *X* and *Y*.

Note that under the more general case, this model can be parameterized by a vector of transition probabilities $\theta = \left\{p(a|w_x,w_y), a \in A, w_x, w_y \in |A|^k \right\}$ taking values in a $(|A|-1)|A|^{2k}$-dimensional space $\Theta$. The parameters in the case of independence (i.e., under the null hypothesis) are in a subset $\Theta _0$ of the parameter space $\Theta$ in which the following restriction holds $\theta = \{p(a|w_x,w_y): p(a|w_x,w_y) = p(a|w_x) \quad \forall w_x, w_y \in |A|^k, a \in A \}$.

A common way to test nested hypothesis is by using the likelihood ratio test. Given the observed data $Y_1^n$, the likelihood ratio test statistics is computed by

16 $$\begin{aligned} R(Y_1^n) = \frac{\sup \{L(Y_1^n;\theta ): \theta \in \Theta _0\}}{\sup \{L(Y_1^n;\theta ): \theta \in \Theta \}}, \end{aligned}$$

where $L(Y_1^n;\theta )$ is the likelihood of the sample $Y_1^n$ for the model specified by $\theta$. This statistics computes a ratio of the maximized likelihood of the sample under each hypothesis. It is known that, under the null hypothesis, as the sample size *n* approaches infinity, the distribution of $-2\log (R_n)$ converges to a $\chi ^2$ distribution with degree of freedom equal to the difference in dimensionality of $\Theta$ and $\Theta _0$. The decision whether or not to reject the null hypothesis is then taken by comparing $-2\log (R(Y_1^n))$ to the $\chi ^2$ value corresponding to a desired statistical significance $\alpha$.

In this case, the statistic is defined by

17 $$\begin{aligned} -2\log (R(Y_1^n))= & {} -2\left[ \sum _{a \in A} \sum _{w_x \in |A|^{k'}} N_n(w_x,a) \log (\hat{q}(a|w_x)) - \right. \nonumber \\{} & {} \left. \sum _{a \in A}\sum _{w_x \in |A|^{k'}}\sum _{w_y \in |A|^{k}} N_n(w_x,w_y,a)\log (\hat{q}(a|w_x,w_y))\right] \end{aligned}$$

where $\hat{q}(a|w_x) = N_n(w_x,a)/ N_n(w_x)$ and $\hat{q}(a|w_x,w_y) = N_n(w_x,w_y,a)/N_n(w_x,w_y)$ are the maximum likelihood estimates of the parameters of the models restricted to $\Theta _0$ and $\Theta$ respectively; and $N_n(w_x,a) = \sum _{i=k'+1}^{n}\mathbbm {1}{\left\{ X_{i-k'}^{i-1} = w_x; Y_i = a\right\} }$, $N_n(w_x,w_y,a) = \sum _{i=k'+1}^{n}\mathbbm {1}{\left\{ X_{i-k'}^{i-1} = w_x; Y_{i-k}^{i-1} = w_y; Y_i = a\right\} }$, $N_n(w_x) = \sum _{a \in A}N_n(w_x,a)$, $N_n(w_x,w_y) = \sum _{a \in A}N_n(w_x,w_y,a)$.

### Supplementary Information

Supplementary Information.

## Data Availability

The data and the codes used in the analyses are available upon request to Claudia D. Vargas, email: cdvargas@biof.ufrj.br
