# Supplementary material for: Probabilistic prediction and context tree identification in the Goalkeeper game
Source: Sci Rep. 2024 Jul 5;14:15467. doi: 10.1038/s41598-024-66009-w (PMC11226460; doi:10.1038/s41598-024-66009-w)
Supplement: Supplementary file 1 — Supplementary Information. [file 41598_2024_66009_MOESM1_ESM.pdf]

Probabilistic prediction and context tree identification in the Goalkeeper Game

Noslen Hernández[1], Antonio Galves[2,**], Jesús E. Garcia[3], Marcos Dimas Gubitoso[2,**], and Claudia D. Vargas[4,*]

[1]INTHERES, Université de Toulouse, INRAe, ENVT, Toulouse, France
[2]Instituto de Matemática e Estatística, Universidade de São Paulo, São Paulo, Brazil
[3]Universidade Estadual de Campinas, Campinas, Brazil
[4]Instituto de Biofísica Carlos Chagas Filho, Universidade Federal do Rio de Janeiro, Rio de Janeiro, Brazil
[*]cdvargas@biof.ufrj.br
[**]In memoriam

1 Normalized proportion of correct predictions

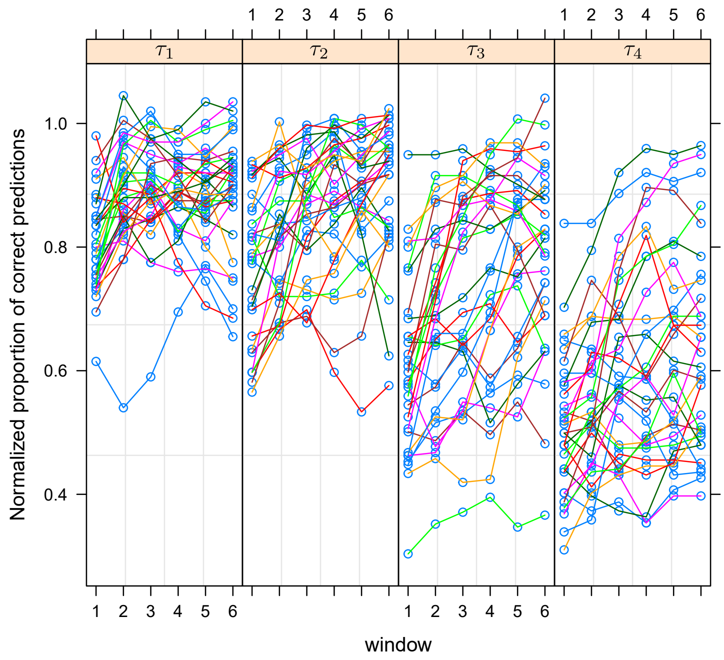

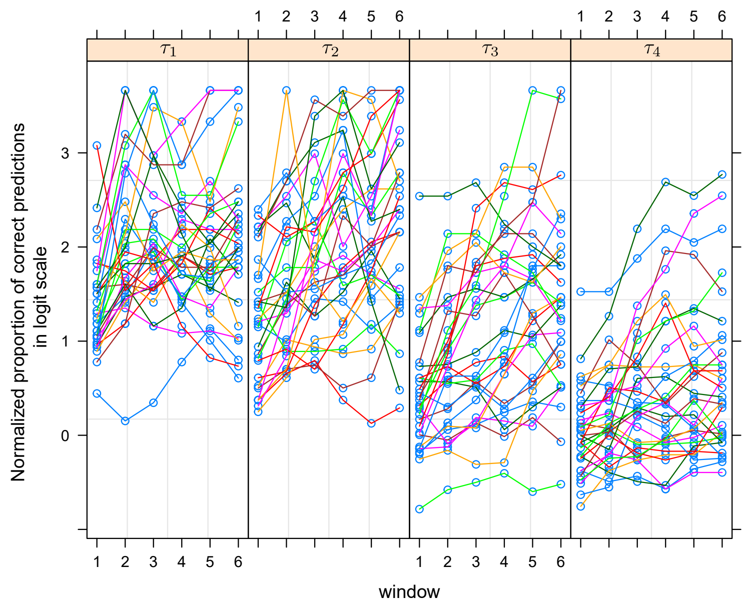

Supplementary Figure S1. (Top) Normalized proportion of correct predictions per time window across participants grouped by context tree model. (Bottom) Normalized proportion of correct predictions after a logit transformation per time window across participants grouped by context tree model.

2 Detecting low compliance

Due to the COVID19 pandemic, we opted to perform remote data collection using a web version of the Goalkeeper game. Compliance measures were integrated into the videogame to ensure essential task completion conditions (e.g., the game running in full-screen mode, termination of the experiment upon exiting the mode, and participants being prohibited from repeating the experiment if they aborted it). Additionally, explicit instructions were provided regarding the importance of avoiding distractions during task execution (such as engaging in conversations or using the phone). Notwithstanding, we observed that a small number of goalkeepers per context tree exhibited a reduction in performance rate over time. We thus chose to exclude these participants from the analysis. Our rationale for this decision was to enhance the validity of our conclusions by removing data points affected by variable compliance, environmental distractions, and other potential factors inherent to the remote context, thus enabling a more accurate reflection of the effects of the experimental protocol design. The exclusion criterion was to remove participants with a negative slope of the least squares fit of the logit-transformed normalized proportion of correct guesses against the time window (Supplementary Figure S2) .

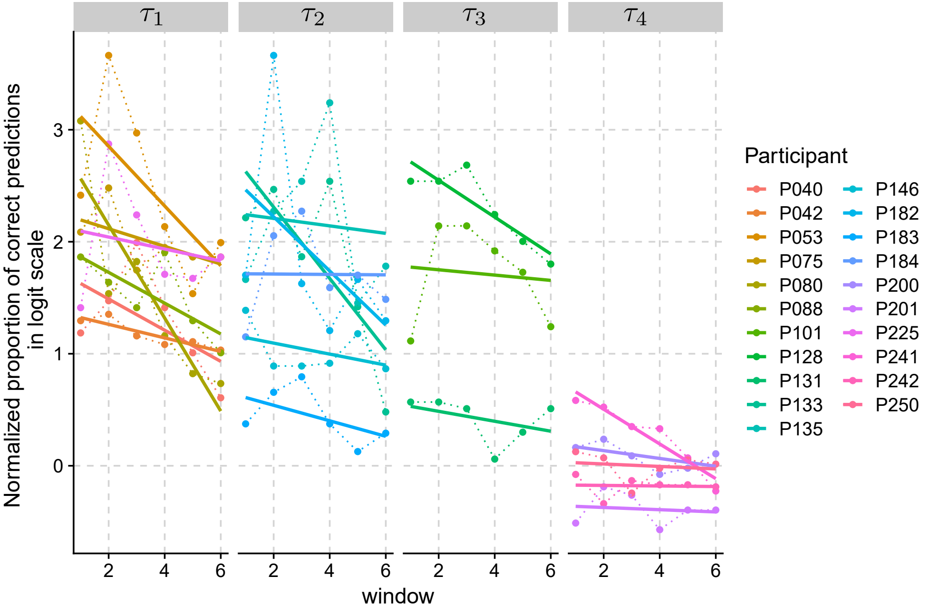

Supplementary Figure S2. Least square fit of the logit-transformed normalized proportion of correct guesses against time window for the participants excluded from the data analysis grouped by context tree model.

To test whether the decrease in performance was significant across time window for the excluded participants, for each context tree model, a non-parametric one-way repeated measure ANOVA (Analysis of Variance of Aligned Rank Transform Data [1]) was conducted. For the models τ_1, τ_2 and τ_3, significant differences were detected across time windows, indicating performance deterioration over time (Supplementary Tables S1, S2 and S3, respectively). No

significant differences were detected across time windows for the participants excluded in τ_4 (Supplementary Table S4). Notably, the match rate achieved by these participants in all time windows fell within the range of match rates obtained when using an strategy known as "random guess" (i.e., when the participant is choosing a symbol uniformly and with equal probability, without any selection criteria).

Effect	Df	Df.res	F	p.value
window	5	30	4.1461	0.0055547 **

Supplementary Table S1. Analysis of Variance of Aligned Rank Transform Data for the excluded participants in model τ_1. Significance codes: 0 "***" 0.001 "**" 0.01 "*" 0.05 "·" 0.1 " " 1

Effect	Df	Df.res	F	p.value
window	5	25	2.4725	0.059589 ·

Supplementary Table S2. Analysis of Variance of Aligned Rank Transform Data for the excluded participants in model τ_2. Significance codes: 0 "***" 0.001 "**" 0.01 "*" 0.05 "·" 0.1 " " 1

Effect	Df	Df.res	F	p.value
window	5	10	2.5918	0.093819 ·

Supplementary Table S3. Analysis of Variance of Aligned Rank Transform Data for the excluded participants in model τ_3. Significance codes: 0 "***" 0.001 "**" 0.01 "*" 0.05 "·" 0.1 " " 1

Effect	Df	Df.res	F	p.value
window	5	20	1.1493	0.36771

Supplementary Table S4. Analysis of Variance of Aligned Rank Transform Data for the excluded participants in model τ_4. Significance codes: 0 "***" 0.001 "**" 0.01 "*" 0.05 "·" 0.1 " " 1

3 Additional ANOVA results

Effect	df	MSE	F	ges	p.value
(Intercept)	1, 97	2.61	423.45 ***	.769	<.001
CTM	3, 97	2.61	31.51 ***	.427	<.001
window	2.74, 265.67	0.30	94.47 ***	.187	<.001
CTM:window	8.22, 265.67	0.30	3.04 **	.022	.003

Supplementary Table S5. ANOVA results with the Sphericity−corrected degrees of freedom (df). The Greenhouse−Geisser method was used for correction. Significance codes: 0 “***” 0.001 “**” 0.01 “*” 0.05 “.” 0.1 “ ” 1

contrast	estimate	SE	df	t.ratio	p.value
τ_1 w1 - τ_2 w1	0.05842	0.1552	97	0.376	0.7467
τ_2 w1 - τ_3 w1	0.85649	0.1508	97	5.679	<.0001
τ_3 w1 - τ_4 w1	0.29188	0.1477	97	1.976	0.0807
τ_1 w2 - τ_2 w2	0.44031	0.1908	97	2.308	0.0418
τ_2 w2 - τ_3 w2	0.83390	0.1854	97	4.498	0.0001
τ_3 w2 - τ_4 w2	0.43548	0.1816	97	2.398	0.0349
τ_1 w3 - τ_2 w3	0.23345	0.2231	97	1.046	0.3774
τ_2 w3 - τ_3 w3	0.95935	0.2168	97	4.425	0.0001
τ_3 w3 - τ_4 w3	0.44551	0.2123	97	2.098	0.0636
τ_1 w4 - τ_2 w4	-0.13317	0.2425	97	-0.549	0.6343
τ_2 w4 - τ_3 w4	1.06382	0.2357	97	4.513	0.0001
τ_3 w4 - τ_4 w4	0.51050	0.2309	97	2.211	0.0507
τ_1 w5 - τ_2 w5	-0.02518	0.2348	97	-0.107	0.9395
τ_2 w5 - τ_3 w5	0.83567	0.2282	97	3.662	0.0013
τ_3 w5 - τ_4 w5	0.67852	0.2235	97	3.036	0.0090
τ_1 w6 - τ_2 w6	-0.29728	0.2474	97	-1.202	0.3046
τ_2 w6 - τ_3 w6	1.15480	0.2404	97	4.803	0.0001
τ_3 w6 - τ_4 w6	0.69589	0.2355	97	2.955	0.0095
τ_1 w1 - τ_1 w2	-0.69603	0.0778	97	-8.942	<.0001
τ_2 w1 - τ_2 w2	-0.31413	0.0778	97	-4.036	0.0004
τ_3 w1 - τ_3 w2	-0.33672	0.0734	97	-4.589	0.0001
τ_4 w1 - τ_4 w2	-0.19313	0.0748	97	-2.583	0.0226
τ_1 w2 - τ_1 w3	-0.18970	0.0976	97	-1.945	0.0832
τ_2 w2 - τ_2 w3	-0.39657	0.0976	97	-4.065	0.0004
τ_3 w2 - τ_3 w3	-0.27113	0.0920	97	-2.948	0.0095
τ_4 w2 - τ_4 w3	-0.26109	0.0937	97	-2.786	0.0144
τ_1 w3 - τ_1 w4	0.05230	0.0826	97	0.633	0.6082
τ_2 w3 - τ_2 w4	-0.31431	0.0826	97	-3.805	0.0009
τ_3 w3 - τ_3 w4	-0.20985	0.0779	97	-2.694	0.0176
τ_4 w3 - τ_4 w4	-0.14485	0.0794	97	-1.825	0.1039
τ_1 w4 - τ_1 w5	-0.10243	0.0799	97	-1.281	0.2756
τ_2 w4 - τ_2 w5	0.00556	0.0799	97	0.070	0.9447
τ_3 w4 - τ_3 w5	-0.22259	0.0754	97	-2.954	0.0095
τ_4 w4 - τ_4 w5	-0.05458	0.0768	97	-0.711	0.5688
τ_1 w5 - τ_1 w6	-0.11111	0.0813	97	-1.367	0.2458
τ_2 w5 - τ_2 w6	-0.38320	0.0813	97	-4.716	0.0001
τ_3 w5 - τ_3 w6	-0.06408	0.0766	97	-0.836	0.4964
τ_4 w5 - τ_4 w6	-0.04670	0.0781	97	-0.598	0.6159

Supplementary Table S6. Detailed results for the 38 statistical tests done. The Benjamini & Hochberg correction was used to control the level of significance with multiple comparisons.

References

[1] Wobbrock, Jacob O., Leah Findlater, Darren Gergle, and James J. Higgins. The aligned rank transform for nonparametric factorial analyses using only anova procedures. In Proceedings of the SIGCHI conference on human

factors in computing systems, pp. 143-146. 2011.
